# Subtrochanteric Osteotomy in Direct Anterior Approach Total Hip Arthroplasty for Crowe IV Dysplasia—Surgical Technique and Literature Review

**DOI:** 10.1111/os.13996

**Published:** 2024-01-31

**Authors:** Zhiming Lu, Qinghuang Chen, Yiping Lan, Shiwei Xie, Feitai Lin, Eryou Feng

**Affiliations:** ^1^ Department of Arthrosis Surgery Fujian Medical University Union Hospital Fuzhou China; ^2^ Department of Orthopedic Anxi County Hospital Quanzhou China; ^3^ Fuzhou Second Hospital Fuzhou China

**Keywords:** Crowe IV hip dysplasia, Direct anterior approach, Literature review, Subtrochanteric osteotomy, Total hip arthroplasty

## Abstract

For Crowe IV dysplasia, the clinical efficacy and surgical technique of subtrochanteric osteotomy (SO) within the direct anterior approach total hip arthroplasty (DAA‐THA) was a subject of debate. This study aimed to describe the surgical technique and clinical outcomes in 11 cases of SO in DAA‐THA and to summarize the relevant literature on this topic. Between June 2016 and June 2023, we retrospectively evaluated patients diagnosed with Crowe IV hip dysplasia at our institution. Criteria identified 11 patients who underwent SO during DAA‐THA. Comprehensive data encompassing demographic information, radiological data, prosthetic implant type, and surgical intricacies were collected. In addition, an exhaustive review of existing case series literature was undertaken utilizing the PubMed databases. There were no revisions, deaths, dislocations, or infections. One hip (9.09%) had an intraoperative proximal split fracture, two hips (18.2%) had lower limb deep vein thrombosis, and one hip (9.09%) had symptoms of femoral nerve injury. Radiological data showed improved bilateral femoral offset, leg length discrepancy, and anatomical acetabular. During the mean follow‐up of 2.18(1.06‐2.46) years, patients demonstrated enhanced functional outcomes, with average changes of 25.2 in the Harris hip score and 47 in the WOMAC score. Reviewing the literature, most studies have favored S‐ROM prostheses and transverse osteotomy techniques. Intraoperative fractures were notably frequent, with rates peaking at 25%. Nonunion and nerve injury were secondary common complications. SO via DAA‐THA may offer satisfactory clinical and radiographic outcomes, but the literature review underscores the need for heightened awareness of intraoperative fracture risk. Proximal detachment of the vastus intermedius plays a pivotal role in SO exposure through the DAA.

## Graphical abstract

We illustrated the practical application of the subtrochanteric osteotomy technique in direct anterior approach total hip arthroplasty. Proximal detachment of the vastus intermedius played a pivotal role in SO exposure through the DAA. (A) proximal femoral release; (B) exposure of the osteotomy site –VI is vastus intermedius and VL is vastus lateralis; (C) reaming the medullary canal –TFL is tensor fasciae latae; (D) marking the osteotomy site and cerclage wire ligation; (E) transverse subtrochanteric shorting osteotomy is completed; (F) reduction of the osteotomy and implantation of the S‐ROM prosthesis.

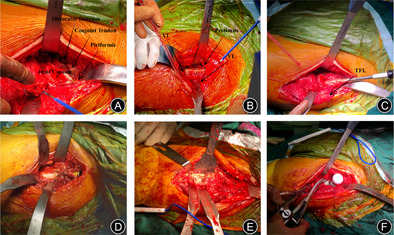

## Introduction

Crowe IV represents the most severe form of developmental dysplasia of the hip (DDH), a condition rare in the general population. It is characterized by a constellation of deformities including complete femoral head dislocation, anatomically hypoplastic acetabulum, and pronounced femoral neck anteversion.^1^ In clinical practice, subtrochanteric osteotomy (SO) has emerged as a consistent approach within total hip arthroplasty (THA) for addressing these complex deformities, rectifying leg length discrepancy (LLD), and mitigating the potential risk of sciatic nerve injury.^2^, ^3^, ^4^, ^5^, ^6^

The direct anterior approach (DAA) in THA, characterized by a less invasive trajectory between neural interface and muscular gap, offers advantages such as reduced soft tissue damage, minimal bleeding, lower dislocation risk, and quicker recovery compared with other approaches.^7^ Simultaneously, DAA‐THA can expose the acetabulum, allowing surgeons to directly visualize and manually confirm the anatomical positioning of the cup.^8^ However, due to limited experience, constrained femoral exposure, and challenges associated with direct stem implantation, the posterior lateral approach has traditionally prevailed over the DAA for SO in Crowe IV DDH patients.^9^, ^10^, ^11^

Currently, SO reports under the DAA‐THA are extremely limited. Oinuma *et al*.^12^ published the first series of DAA‐THA combined with SO for Crowe IV DDH patients (12 hips) in 2014, with positive preliminary clinical outcomes. There was, however, a lack of detail on surgical techniques and intraoperative anatomy. In addition, researchers including Liu *et al*.^5^, ^13^, ^14^ and Viamont‐Guerra *et al*.^15^, ^16^ also explored the clinical efficacy of SO for hip dysplasia via DAA, and provided relevant experience on the surgical techniques and difficulties. Nonetheless, limited research has resulted in controversies and gaps in clinical benefits and surgical techniques. There was a particular lack of anatomical understanding and discussion regarding the technical challenge of subtrochanteric osteotomy exposure via the DAA.^2^, ^9^ Moreover, to our knowledge, no existing literature summarizes information on prosthetic selection, complications, and osteotomy related to SO in DAA‐THA. As a result, there was a significant gap in understanding the efficacy and potential complications of this surgical strategy.

Through cases analysis conducted at our institution's joint center and a comprehensive review of relevant literature, our goals were to: (i) evaluate the early clinical and radiographic results of SO in DAA‐THA in a group of Crowe‐IV DDH patients (11 cases); (ii) provide our experience and description of the surgical technique, especially new insights on the exposure of SO under the DAA; and (iii) review and summarize the understanding of complications, prosthesis segregation, and prosthesis placement.

## Methods and Surgical Techniques

### Participant Selection

The study protocol was based on the principles of the Declaration of Helsinki with the informed consent of all patients, and approved by the medical ethics committee of Fuzhou City Second Hospital (grant no. 2022083). The inclusion criteria were as follows: (i) meeting diagnostic criteria for congenital Crowe IV DDH, characterized by typical clinical presentations and X‐ray manifestations; (ii) undergoing primary DAA‐THA; (iii) implementation of SO technique during surgery; (iv) availability of preoperative and postoperative anteroposterior (AP) and lateral pelvic radiographs; and (v) minimum one‐year follow‐up duration after osteotomy. Exclusion criteria comprised: (i) patients lacking complete clinical data; (ii) incomplete follow‐up data; (iii) intraoperative alteration of surgical approach; (iv) prior affected hip surgeries; and (v) presence of debilitating postoperative recovery‐affecting conditions (e.g., poliomyelitis, Parkinson's disease). Following strict inclusion and exclusion criteria, our final cohort comprised 11 patients (11 hips), with an average follow‐up period of 2.18 (1.06–2.46) years.

All surgical procedures were performed within our institution by the senior author. Cementless hemispheric porous‐coated acetabular cups, modular S‐ROM (DePuy, Raynham, MA, USA) prostheses and Wagner Cone (DePuy, Raynham, MA, USA) were used in SO. Preoperative three‐dimensional surgical planning was facilitated through the utilization of the artificial intelligence hip software (AIHIP, version 3.0, Longwood Valley Technology, Beijing, China), grounded in preoperative pelvic CT scans. This software harnessed artificial intelligence algorithms to formulate 3D preoperative plans and provide implant types.^17^ During surgery, adaptations to the preoperative 3D plans were implemented based on our clinical experience.

### Data Collection

Scrutinizing patient medical records yielded a comprehensive dataset comprised of demographic information (age, gender, BMI), key procedural details, intraoperative blood loss, creatine kinase fluctuations, implant specifications, perioperative complications, surgical duration, osteotomy length and length of hospital stay. Primary assessments of patient‐reported outcomes included the Harris hip score and the Western Ontario and McMaster Universities Osteoarthritis Index (WOMAC). The radiographic dataset involved metrics including leg length discrepancy, hip center of rotation, acetabular abduction and anteversion angles and femoral offset. Follow‐up durations and any revision surgery instances were recorded. The work of Shen *et al*.^18^ offers explicit guidelines for radiographic measurements. Intraoperative blood loss was calculated by using the Cross method: intraoperative blood loss = (preoperative erythrocyte volume − postoperative erythrocyte volume) / mean erythrocyte volume.^19^, ^20^ In addition, osteotomy‐related data, encompassing osteotomy length, nonunion occurrences, and osteotomy healing time were collected.^21^, ^22^ The article also introduced the surgical procedure depiction, which was complemented by a practical case illustration. Two highly trained and experienced orthopedic surgeons measured all radiographic assessment parameters after having received rigorous training. Statistical analysis was performed using the arithmetic mean of both measurements.

### Surgical Technique

#### Surgical Incision

The standard DAA incision was extended both proximally and distally. The incision extended downwards more than upwards, while the incision center was maintained relative to the anterior superior iliac spine. Layers of skin, subcutaneous tissue and fascia along the surgical incision were dissected. The proximal portion was detached from the anterior superior iliac spine and tagged for subsequent repair at the conclusion of the procedure as a means of preventing traction‐related injury to the tensor fasciae late. After accessing the Heuter space, the ascending branch of the lateral circumflex femoral artery was identified before being subjected to electrocoagulation. Traversing the anterior aspect of the femoral neck capsule, the retroflexed head of the rectus femoris muscle was discerningly identified. The maneuver was tailored to the extent of soft tissue contracture and a methodical release of the retroflexed head of the rectus femoris muscle then occurred. The lower limb was subsequently externally rotated, enabling a meticulously executed H‐shaped resection of the anterior and superior aspects of the articular capsule.

#### Acetabular Component Implantation

**FIGURE 1:**
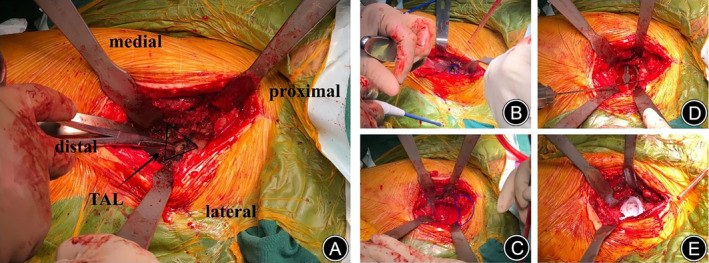
Surgical operation diagram: (A) locating the anatomical acetabulum—Δ represents triangular fossa and TAL is transverse acetabular ligament; (B) removal of osteophyte above the acetabulum; (C) acetabular apex deficiency following reaming; (D) autologous femoral head graft; (E) acetabular prosthesis implantation.

With a slight external rotation of the affected limb, the femoral neck was transected approximately 0.5–1.0 cm above the lesser trochanter, and the femoral head was subsequently extracted. In order to prevent damage to the greater trochanter, we prefer to utilize a reciprocating saw for bone cutting in the vertical plane of the femoral neck, with a slight inclination toward the lesser trochanter. We were able to locate the true acetabulum by identifying the termination insertion of the anterior capsular. Recognizing the triangle‐shaped fossa beneath the pseudoacetabulum and the transverse ligament of the acetabulum at the base of the acetabular fossa aids in delineating both the upper and lower margins of the anatomical acetabulum (Figure 1A **)**. After performing adequate capsular release, it was imperative to meticulously remove osteophytes in the vicinity of the acetabulum, particularly those located at the superior rim (Figure 1B). Acetabular reaming was guided by the superior margin of the acetabulum. Vertical grinding was performed using a 36–38 mm acetabular reamer, and the depth was determined by the inner border of the ilium. Sequential grinding was then carried out posteriorly and superiorly, paying close attention to protecting the anterior wall (Figure 1C). In cases where the degree of bone deficiency at the acetabular cup's superior rim exceeded 30%, selective usage of autologous femoral head graft or tantalum metal wedge was taken into consideration (Figure 1C,D). Acetabular reamers in the sizes 44, 46, and 48 mm were more commonly utilized. The ideal acetabular abduction angle was 40° ± 5°, and the anteversion angle ranged from 20° to 25°. After that, a biological‐type acetabular prosthesis was securely implanted, affixed with two or three screws, and accompanied by the placement of a ceramic or polyethylene liner (Figure 1E).

#### Subtrochanteric Osteotomy and Stem Implantation Technique

**FIGURE 2:**
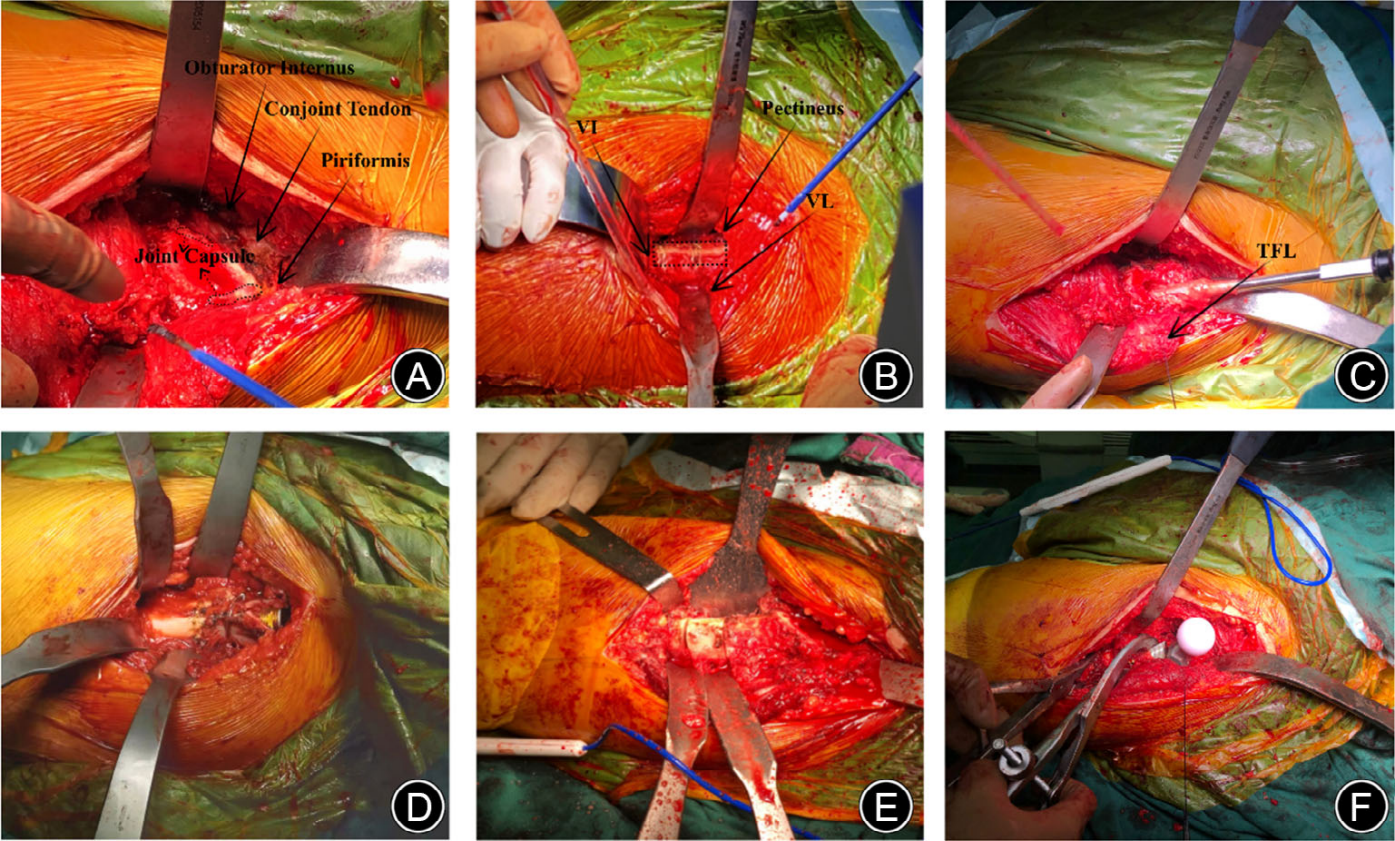
Surgical operation diagram: (A) proximal femoral release; (B) exposure of the osteotomy site—VI is vastus intermedius and VL is vastus lateralis; (C) reaming the medullary canal—TFL is tensor fasciae late; (D) marking the osteotomy site and cerclage wire ligation; (E) transverse subtrochanteric shorting osteotomy is completed; (F) reduction of the osteotomy and implantation of the S‐ROM prosthesis.

**FIGURE 3:**
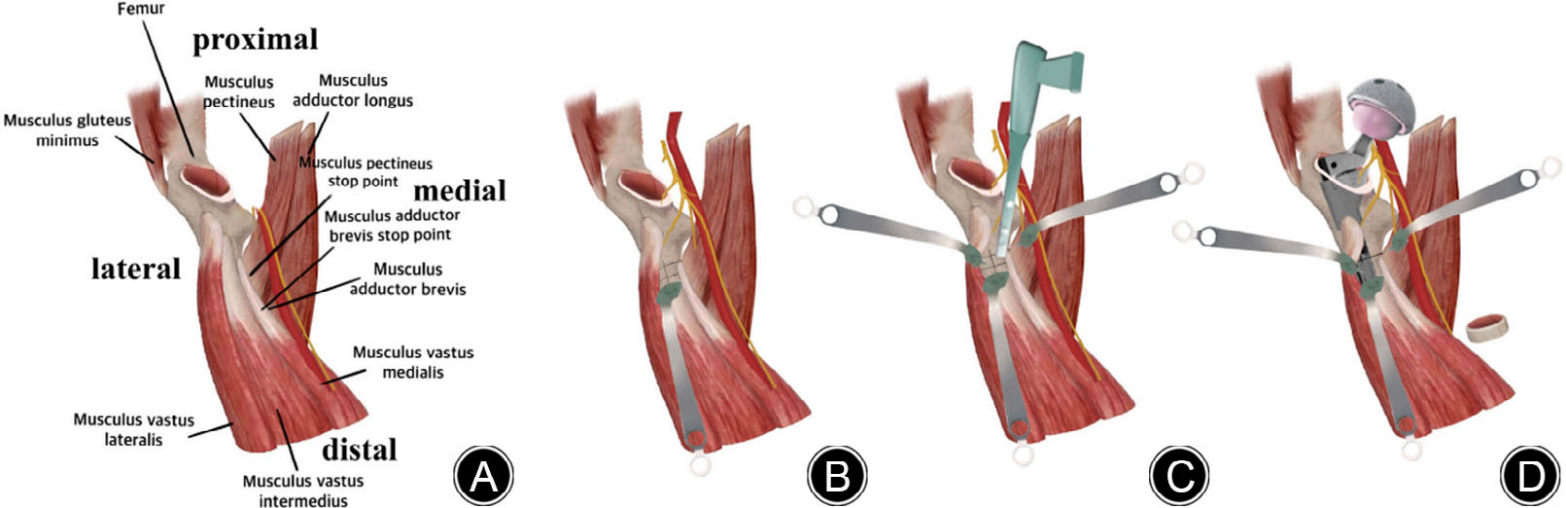
Anatomical schematic: (A) the affected limb in internal and external rotation; (B) exposure of the osteotomy site; (C) transverse osteotomy procedure; (D) removing the truncated bone block and re‐implanting the SROM prosthesis pilot component to reset.

When it comes to proximal femoral release, we typically opt for releasing the medial and lateral joint capsules, the piriformis muscle, and the conjoint tendon, while at the same time, preserving the external rotators (Figure 2A). By excessively internally rotating and externally rotating the affected limb, we elevated the femur, and proximally dissected the vastus intermedius and vastus lateralis so as to expose the pectineus muscle as well as the osteotomy site (Figure 2B). A modular S‐ROM prosthesis was used in the majority of procedures, and standard proximal and distal reaming techniques were used to adhere to modular design guidelines (Figure 2C). Marking the osteotomy site with a proximal sleeve, we pre‐tied a cerclage wire to prevent fractures (Figure 2D). Based on the preoperative AIHIP planning, leg length discrepancy, and the degree of intraoperative hip joint reposition, we performed one to three osteotomies around the pectineus, if necessary, gradually increasing the amount of osteotomy (Figure 2E). Typically, the amount of osteotomy should not exceed the disparity in the lower limbs, and the osteotomy line generally not extend beyond 8 cm below the greater trochanter, as such actions could potentially impact the stability of the prosthesis.^23^ The osteotomy blocks were then excised, the osteotomy site was realigned, and the void was filled using autograft obtained from acetabular reaming. The optimal osteotomy gap was maintained below 1 mm. Finally, the femoral stem was inserted into the sleeve, and the anteversion angle was adjusted for the purpose of achieving optimal alignment (Figure 2F). The anatomical simulation of the osteotomy site was illustrated in Figure 3.

#### Postoperative

After the surgery, a pillow was positioned in the popliteal fossa to mitigate excessive stress on the sciatic nerve. Intravenous antibiotics were administered for 24 h in combination with a multimodal analgesia regimen and oral anticoagulants that lasted for 3–5 days. A postoperative drain was inserted, which was then removed on postoperative day (POD) #1. Weight‐bearing was discouraged during the initial week and partial weight‐bearing with the assistance of a walker was advised during weeks 2–4.

#### Literature Review

We used the keywords “Dysplasia” and “Subtrochanteric Osteotomy” to search the PubMed database from 1987 to 2023. This search yielded 174 studies that were potentially relevant. We excluded research involving the posterior lateral approach, studies with duplicated populations from the same institution, case reports, and those focusing on lower‐grade (Crowe I‐II dislocation) DDH during the screening process. Finally, our review of the literature included only five articles that were specifically about SO combined with DAA‐THA.

#### Statistical Analysis

Descriptive data were presented as mean values accompanied by standard deviations (SD) or as frequencies and percentages. Reliability between raters and within raters was assessed using the intraclass correlation coefficient (ICC). Radiographic evaluations of prosthesis positioning were performed both pre‐ and post‐surgery and the rotation center was assessed following hip joint reconstruction. These were subjected to paired‐design data *t*‐tests for analysis. A *p*‐value of < 0.05 was regarded as being statistically significant.

## Results

### Perioperative Clinical Data

A total of 11 patients (three males and eight females) participated in this study. Their average age was 50.27 years and the mean BMI was 22.69 kg/m^2^. The surgeries took an average of approximately 156.18 min and patients spent an average of 7.91 days in hospital following their operations. Operative complications included femoral fractures, deep vein thrombosis and femoral nerve injuries. The average creatine kinase level discrepancy was 1008.00 U/L and blood loss was 901.92 mL. Average osteotomy was 22.50 mm. All 11 patients experienced delayed union and there were no nonunion cases. Osteotomy healing took 6.34 months on average. In nine cases, the S‐ROM prosthesis was used and the Wagner Cone was used in two cases. The specific clinical data and prosthesis information can be seen in Table 1.

**TABLE 1:** Perioperative clinical data.

	*N* or mean (SD)
Gender (male/female)	3/8
Affected side (right/left)	3/8
BMI (kg/m^2^)	22.69 ± 2.53
Follow‐up (years)	2.18 ± 0.48
Age (years)	50.27 ± 14.87
Operative duration (min)	156.18 ± 38.37
Post‐op LOH (days)	7.91 ± 3.62
Operative complications	4
Intraoperative fracture	1
DVT	2
Femoral nerve injury	1
Creatine kinase (D1–0) (U/L)	1008.00 ± 576.69
Intraoperative blood loss (mL)	901.92 ± 395.41
Osteotomy length (mm)	22.50 ± 12.82
Osteotomy nonunion	0
Delayed union	11
Osteotomy healing time (months)	6.34 + 0.77
Prosthesis type	
S‐ROM	9
Wagner Cone	2
Cup size (mm)	
44	1
46	3
48	6
50	1
Head size (mm)	
28	2
32	8
36	1

Abbreviations: D1–0, the difference of creatine kinase between 1 day after surgery and before surgery; DVT, deep vein thrombosis; LOH, length of hospitalization; post‐op, post‐operative.

### Clinical Assessment and Radiographic Measurement

The preoperative and postoperative femoral offset difference was measured and compared with the healthy side and changes in the rotation centers of bilateral hip joints were also measured. These measurements were then subjected to rigorous statistical analysis. Radiological assessment was used to evaluate the effectiveness of anatomical acetabular reconstruction. Specific details are presented in Table 2, which reveal statistically significant differences. There was significant improvement to both average WOMAC and Harris scores. The former decreased from 66 to 19, while the latter increased from 33.00 to 58.2.

**TABLE 2:** Radiological data variation results (compared to the healthy side).

	Preoperative	Postoperative	T value	*p* value
LLD, mean ± SD	29.50 ± 14.06	7.00 ± 5.18	10.11	<0.001
Femoral offset difference, mean ± SD (mm)	13.59 ± 7.70	6.50 ± 4.25	4.14	0.002
HCOR, mean ± SD				
Level difference (mm)	17.28 ± 8.24	5.23 ± 3.77	4.30	0.002
Vertical difference (mm)	42.61 ± 23.26	5.32 ± 3.85	5.25	<0.001

Abbreviations: HCOR, hip center of rotation; LLD, leg length discrepancy; SD, standard deviation.

### Case Presentation

**FIGURE 4:**
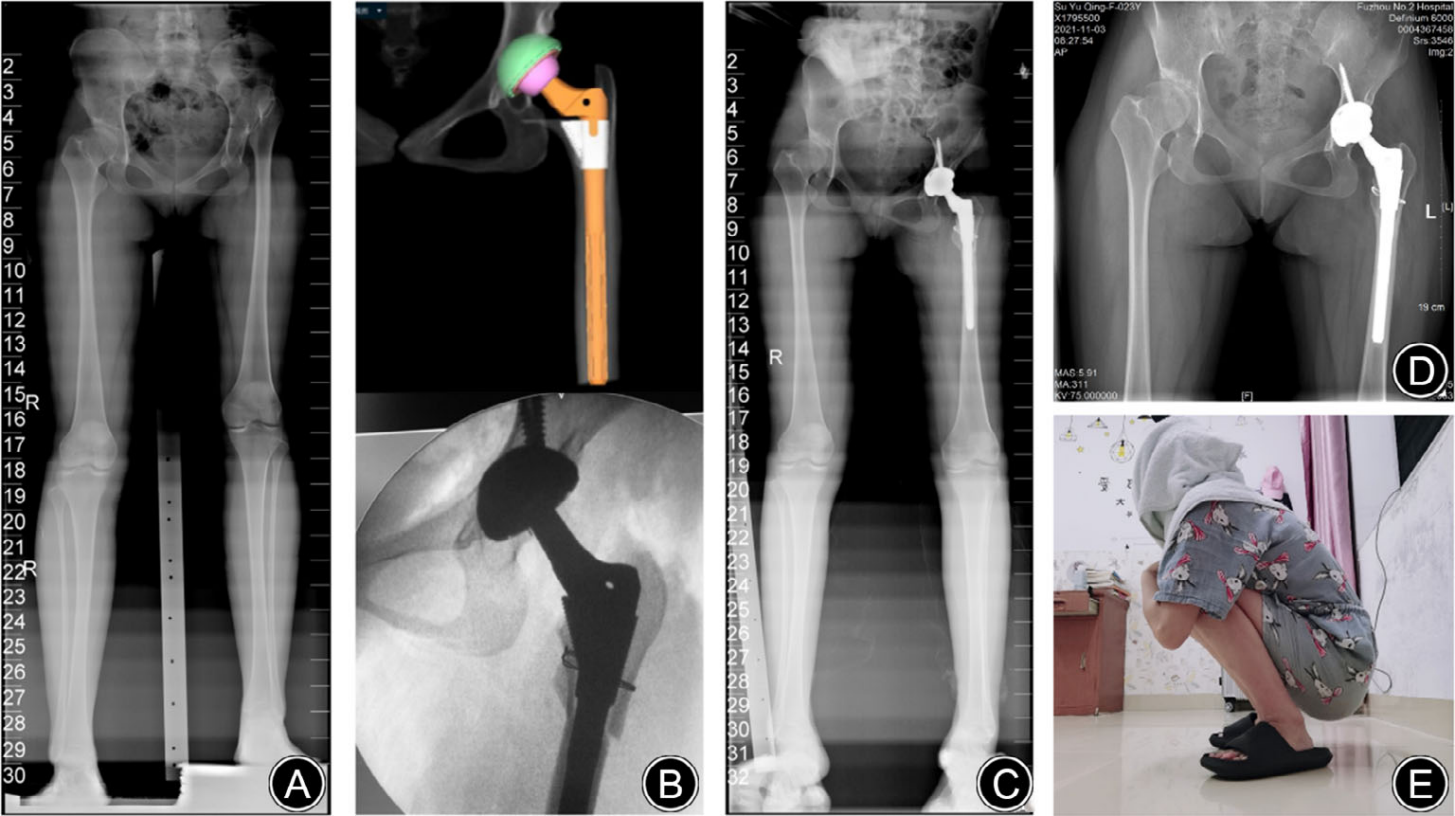
Case presentation: DAA‐THA for unilateral hip dysplasia in 22‐year‐old female: (A) preoperative full‐length X‐rays of the lower limb; (B) the comparison between preoperative AI planning and intraoperative X‐ray fluoroscopy; (C) postoperative full‐length X‐rays of the lower limb; (D) anteroposterior radiograph of pelvis 1 year after operation; (E) the hip activity at last follow‐up.

A 22‐year‐old female with a body mass index of 19.78 kg/m^2^ presented with a history of chronic progressive left hip pain and a limp she had had for a decade, which led to her admission to our clinic in December 2019. Subsequent imaging included an anteroposterior (AP) radiograph of the pelvis and a full‐length radiograph of the lower limb. This revealed Crowe type IV DDH, which was characterized by complete dislocation and a leg length discrepancy measuring 34.37 mm. Surgical intervention was required for implanting a 44 mm cementless porous‐coated acetabular cup, paired with a modular S‐ROM stem measuring 16 × 11 × 150 mm, supported by two acetabular screws and a cable for additional stability. The preoperative, intraoperative and postoperative X‐rays are shown in Figure 4. The osteotomy healed completely after approximately 4.80 months. At the final follow‐up, the Harris score of the patient was 82 and the WOMAC score was 13.

## Discussion

Performing SO in THA for Crowe IV DDH is technically demanding and did present many challenges to the surgeon in terms of both the femoral and acetabular sides.^5^, ^22^, ^24^ The feasibility of SO with DAA‐THA depends on surgeon expertise, making it relatively contraindicated. Only limited reports discussed the use of SO within DAA for Crowe III or IV DDH (Table 3). This study elaborated on the SO procedure in DAA‐THA, supplemented by the favorable results of a case series. Based on clinical and radiological outcomes, our findings indicated that this procedure was both safe and effective. In terms of the technical difficulty of SO exposure, we believed that detaching the vastus intermedius muscle was critical for obtaining the osteotomy window. This maneuver was thought to help achieve a clear and defined surgical interface.

**TABLE 3:** Literature review on clinical outcomes and complications of THA via DAA (HAA) for severe hip dysplasia (Crowe III‐IV) with SO (hips *n* > 1)

Reference	Year	Surgical approach	Crowe type	Cohort	Age	Osteotomy		SO (%)	Prosthesis (femoral stems)	LLD (mm, mean)		Osteotomy nonunion	Operative complications (*n*)		Revisio*n* (%)
				Hip (*n*)	Mean	Location	Method			Pre	Post		Intra	Post	
This study	2022	DAA	IV	11	50	Pectineus insertion	Transverse	100.0%	S‐ROM+ Wagner Cone	29.5	7.0	1	1 PFF	2 DVT/ 1 Femoral nerve injury	0
Zhang et al.^13^	2020	DAA	III‐IV	15 (8 III / 9 IV)	25	10–15 mm (SO)	Transverse	33.30%	S‐rom + Trilock	41.0	5.0	0	2 DFF/ 4 TFL injury/ 3 LCFN injuries		0
Viamont‐Guerra et al.^16^	2020	HAA	IV	8	44	10 mm (SO)	Transverse	62.5%	‐	22.9	3.2	1	1 FF / 1 *CF*	1 SO nonunion	0
Viamont‐Guerra et al.^15^	2020	DAA	III‐IV	29 (20III/9IV)	49	Preoperative planning	‐	17.2%	‐	25.0	3	1	2 FF /1 FSTC /1 *CF*	1 TFF injury / 1 malunion / 2 loosening / 1 PE wear + osteolysis	24.1%
Yildirim *et al*.^41^	2015	DAA	I‐IV	102 (22 III + 39IV)	49	Preoperative planning	‐	59.8%	Zweymuller + PPF + CDH	Improved		3	25		14.7%
Oinuma *et al*.^12^	2014	DAA	IV	12	62	Preoperative planning	Transverse + Oblique	100.0%	S‐ROM + BiCONTACT	35 (Net improvement)		0	–	1 dislocation	0

*Note*: The literature review does not include case reports.

Abbreviations: (P/D) FF, (Proximal/Distal) Femoral Fracture; *CF*, Calcar Fracture; DAA, Direct Anterior Approach; DVT, Deep Vein Thrombosis; FSTC, Femoral Shaft Trajectory Correction; HAA, Hueter Anterior Approach; LCFN, Lateral Femoral Cutaneous Nerve; LLD, Leg Length Discrepancy; SO, Subtrochanteric Osteotomy; TFL, Tensor Fasciae Latae; Y, year.

### Clinical Evaluation of SO in DAA‐THA

Our surgical duration, blood loss, and length of hospital stay have all decreased significantly when compared with the initial study reported by Oinuma *et al*.^12^ This improvement could be attributed to a variety of factors, such as advances in preoperative planning tools, cumulative technical experience, and an accelerated postoperative rehabilitation program.^25^, ^26^ In our investigation, postoperative functional outcomes demonstrated a significant enhancement in patients' rehabilitation. When compared with preoperative values, radiological analyses revealed statistically significant improvements in bilateral femoral offset discrepancy, LLD, and hip center of rotation. These observed improvements contribute to the restoration of biomechanical alignment and joint stability, which is consistent with previous research findings.^13^ On the other hand, despite the favorable clinical outcomes achievable with the posterior lateral approach in THA for high‐dislocation DDH, some limitations persist, such as postoperative limping and weakened abductor muscle strength. The DAA for treating high‐dislocation DDH resulted in improved functional scores, faster recovery of hip abductor and flexor strength, and improved limp recovery when compared to the posterior lateral approach.^14^ This could be attributed to the disruption of the posterior muscle group in the posterior lateral approach, resulting in more extensive muscle damage. It explained some of the smaller changes in our creatine kinase levels.

### Complication Analysis

The literature highlighted concerns, particularly in terms of intraoperative fractures and osteotomy non‐union. These complications could potentially lead to issues such as varus malalignment, pain, instability and prosthetic loosening.^12^, ^24^, ^27^, ^28^, ^29^ Common complications when using the posterior‐lateral approach include acetabular loosening, delayed osteotomy union, nerve injuries and dislocations.^13^, ^30^, ^31^, ^32^, ^33^ However, during the mid‐term follow‐up, no dislocations or nonunions were observed and delayed unions were more frequent, potentially as a result of less extensive soft tissue dissection and trauma in our study. Compared with case series via the posterior lateral approach, the average healing time of the osteotomy in this study was also shorter. Although minimally invasive injuries may provide a better healing environment for the osteotomy site, the majority of osteotomies still experience delayed healing. Kawai *et al*.^34^ reviewed 27 THAs with subtrochanteric transverse shortening osteotomy for Crowe Type IV hips and found there to be a higher risk of delayed union with shorter femoral resection lengths. It is noteworthy that intraoperative fractures during the DAA are not rare, potentially attributed to various factors. On the one hand, the proximal and distal narrowing of the femur in Crowe IV DDH patients may lead to improper implant fitting. In this study, intraoperative fractures resulted from excessive force during the use of a large cuff. On the other hand, the limited exposure provided by the DAA may contribute to tension in the iliacus and external rotator muscles, potentially resulting in subtrochanteric avulsion fractures. Therefore, in proximal femoral exposure, cerclage wires fixation was often employed to prevent split fractures. According to research by Grob *et al*.,^35^ this method may pose a risk to the anterolateral portion of the quadriceps femoris and vital neurovascular structures, potentially explaining the observed nerve injuries.

### Subtrochanteric Osteotomy Technique Analysis

Although various complex osteotomy techniques, including oblique osteotomies, have been employed, a study by Muratli *et al*.^36^ showed that no single inherent feature significantly increased osteotomy design stability. In a meta‐analysis conducted by Li *et al*.,^37^ transverse osteotomy showed there to be no significant differences in complications and survival rates compared with other osteotomy. However, its simplicity was advantageous for the correction of abnormal anteversion angles and the minimization of periosteal damage.^36^ The conventional SO location was typically situated approximately 1–2 cm below the femoral lesser trochanter, which was determined through meticulous preoperative planning. In cases that involved the S‐ROM prosthesis, a secure osteotomy line was identified at a 2 cm distance distal to the cuff.^13^, ^15^, ^16^, ^32^, ^38^ However, ambiguity remained regarding how to expose and localize the osteotomy site based on limb position. Viamont‐Guerra et al.^15^, ^16^ performed SO with the hip externally rotated to resect 10 mm of the femoral diaphysis below the lesser trochanter. Li *et al*.^5^ and Liu *et al*.^13^, ^39^ exposed the osteotomy site by splitting the vastus lateralis between the interval of vastus lateralis and rectus femoris. In comparison to the work of Zhang *et al*., the vastus lateralis muscle insertion shifted more posteriorly and the vastus intermedius and vastus medialis shifted more laterally in this research. It was considered that there were more lateral and anterolateral than medial muscle attachments at the proximal femur and the pectineus was the highest muscle in the medial thigh muscle, with its insertion closest to the lesser trochanter of the femur, which is a traditional safe osteotomy region. During adduction and external rotation of the lower limb, the osteotomy surface was covered by the vastus intermedius muscle. The vastus intermedius muscle was gently detached at the proximal femur, which revealed the subtrochanteric area beneath the lesser trochanter. If the exposed area was insufficient, it would be possible to further split the vastus lateralis muscle. Careful dissection with a bone retractor was the key to avoiding injury to the femoral nerve and protecting the vastus lateralis and vastus intermedius. The planned osteotomy was performed from the proximal insertion. The osteotomy site could be exposed more easily in the supine position in DAA‐THA than in the lateral position.

### Prosthesis Analysis

Of the femoral stems that were utilized in SO, the most frequently reported option was the modular S‐ROM stem.^12^, ^13^, ^32^ Although some studies suggest that the Wagner Cone was more suitable when the proximal femur was relatively slender.^30^ There were no significant differences in long‐term survival rates between these options. The combination of S‐ROM modular prosthesis in severe hip dysplasia patients proved more suitable for filling the femoral canal in comparison to single‐piece prostheses. This facilitated excellent torsional stability at the osteotomy site through the stabilization of the metaphyseal and diaphyseal osteotomy block.^38^, ^40^ Given the limited visual field in DAA, the modular S‐ROM system appeared to offer greater convenience in correcting abnormal femoral anteversion. The literature also reported there to be a trend towards the use of S‐ROM prostheses, which is consistent with the clinical cases in this study.^5^, ^12^, ^13^, ^14^ Additionally, due to the relatively soft bone quality in Crowe IV patients, the conventional approach involved selecting a porous acetabular cup and securing it with two screws.

### Limitations and Prospects of Clinical Application

It is recognized that there are some limitations to this study. Limited sample size and a single center retrospective study that is reliant on the experience of a single surgeon are the main limitations, which could potentially impact the clinical outcome. However, due to the rarity of Crowe IV DDH and technical and experiential limitations, conducting multicenter randomized controlled trials is quite difficult for researchers. Despite inherent limitations, our study offered valuable groundwork and guidance for exploring the feasibility of SO in Crowe IV DDH patients undergoing DAA‐THA. We supplemented and improved Oinuma *et al*.'s^12^ blank area in surgical techniques by providing more detailed surgical techniques and subtrochanteric anatomical exposure. This served as a reference for the standardized surgical procedure of osteotomy for Crowe IV patients using the DAA. Furthermore, by conducting the first literature review on this specific surgical topic, we gained a better understanding of its complications, thereby expanding the knowledge base in this field to some extent.

## Conclusion

The early clinical outcomes of transverse SO in Crowe type IV DDH during DAA‐THA may be favorable but face substantial technical challenges and limited exploration due to minimal experience. The separation of the vastus intermedius muscle plays a role in osteotomy exposure and nerve protection. Existing literature emphasizes the preference for combining transverse osteotomy with the S‐ROM prosthesis. However, in comparison to nonunion and nerve damage, intraoperative fractures appear to occur more frequently, possibly warranting increased awareness.

## Conflict of Interest Statement

The authors declare that the research was conducted in the absence of any commercial or financial relationships that could be construed as a potential conflict of interest.

## Author Contributions

Study design and surgery performed by EF. Data collection and analysis by ZL, QC, FL, YL, and SX. Manuscript preparation by EF, QC, ZL, FL, YL, and SX.

## Data Availability

The datasets used and/or analyzed during the current study are available from the corresponding author on reasonable request; please contact the corresponding author, Dr. Feng.
